# Field Measurements Indicate Unexpected, Serious Underestimation of Mussel Heart Rates and Thermal Tolerance by Laboratory Studies

**DOI:** 10.1371/journal.pone.0146341

**Published:** 2016-02-03

**Authors:** Morgana Tagliarolo, Christopher D. McQuaid

**Affiliations:** Department of Zoology and Entomology, Rhodes University, Grahamstown, South Africa; Seoul National University, REPUBLIC OF KOREA

## Abstract

Attempts to predict the response of species to long-term environmental change are generally based on extrapolations from laboratory experiments that inevitably simplify the complex interacting effects that occur in the field. We recorded heart rates of two genetic lineages of the brown mussel *Perna perna* over a full tidal cycle *in-situ* at two different sites in order to evaluate the cardiac responses of the two genetic lineages present on the South African coast to temperature and the immersion/emersion cycle. “Robomussel” temperature loggers were used to monitor thermal conditions at the two sites over one year. Comparison with live animals showed that robomussels provided a good estimate of mussel body temperatures. A significant difference in estimated body temperatures was observed between the sites and the results showed that, under natural conditions, temperatures regularly approach or exceed the thermal limits of *P*. *perna* identified in the laboratory. The two *P*. *perna* lineages showed similar tidal and diel patterns of heart rate, with higher cardiac activity during daytime immersion and minimal values during daytime emersion. Comparison of the heart rates measured in the field with data previously measured in the laboratory indicates that laboratory results seriously underestimate heart rate activity, by as much as 75%, especially during immersion. Unexpectedly, field estimates of body temperatures indicated an ability to tolerate temperatures considered lethal on the basis of laboratory measurements. This suggests that the interaction of abiotic conditions in the field does not necessarily raise vulnerability to high temperatures.

## Introduction

The assessment of physiological variability under laboratory conditions allows the consideration of the influence of single stressors, without possible interactions among stressors [1,2], but as a result it is difficult to extrapolate to field conditions. In the case of intertidal organisms, physiological responses are complicated because of the alternation of immersion and emersion and the effects of waves. Previous studies performed on limpets showed that heart rate is a simple and practical technique to monitor the physiological responses of intertidal animals under natural conditions [2,3]. The use of the non-invasive infrared technique allows reliable recording of cardiac activity directly on the shore and causes minimum manipulative stress for the animals [2]. Several studies has analysed the cardiac activity of mussels in the laboratory [4–6], but very few have assessed how this translates to physiological responses in the field [7].

The brown mussel, *Perna perna* L. is native to South Africa and recent studies have shown that two genetic lineages (east and west) exist, with different distributions that overlap along approximately 200km of coastline [8]. The two lineages have been recognized as having a non-sister relationship and do not share a common ancestry [9]. Laboratory experiments showed that the two lineages have different tolerances to sand inundation, high temperature and aerial exposure [10,11], but these differences cannot explain their geographic distributions.

Given their different physiological abilities, we wished to assess how they are likely to respond to long-term environmental change. Our understanding of potential changes in species distributions is largely based on laboratory studies of physiology. Previous laboratory comparisons of these lineages indicated no difference in their upper thermal limits, but important differences in the activation energies of Arrhenius plots [10]. We hypothesized that the different thermal responses in cardiac activity measured in the laboratory might be magnified under natural conditions due to the presence of multiple stressful factors. Here, we examine the heart rates of the two lineages of *P*. *perna* under natural conditions for comparison with laboratory measurements of cardiac rates for individuals collected from the same sites [10]. Because there are no known morphological differences between the two lineages, the effect of tidal cycles on cardiac activity was evaluated by repeating the experiment on two different rocky shores outside the region of overlap, in areas where each lineage exists in isolation, under field conditions that each would normally experience. Moreover, the deployment of temperature loggers on the field allowed us to evaluate the magnitude of temperature stress that can occur in nature. This allowed an evaluation of the effectiveness of physiological studies performed under laboratory-controlled environments compared to measurements of the metabolism of animals living under natural conditions.

## Materials and Methods

### Ethics Statement

Only invertebrate marine molluscs (mussels) were employed in this study. The field work was performed under the field permit RES2014/12 for the purposes of scientific investigation or practical experiment in terms of section 83 of the marine living resource act (act no 18 of 1998). The permit was issued by the department of Agriculture, Forestry and Fisheries of the Republic of South Africa.

### Study site

**Fig 1:**
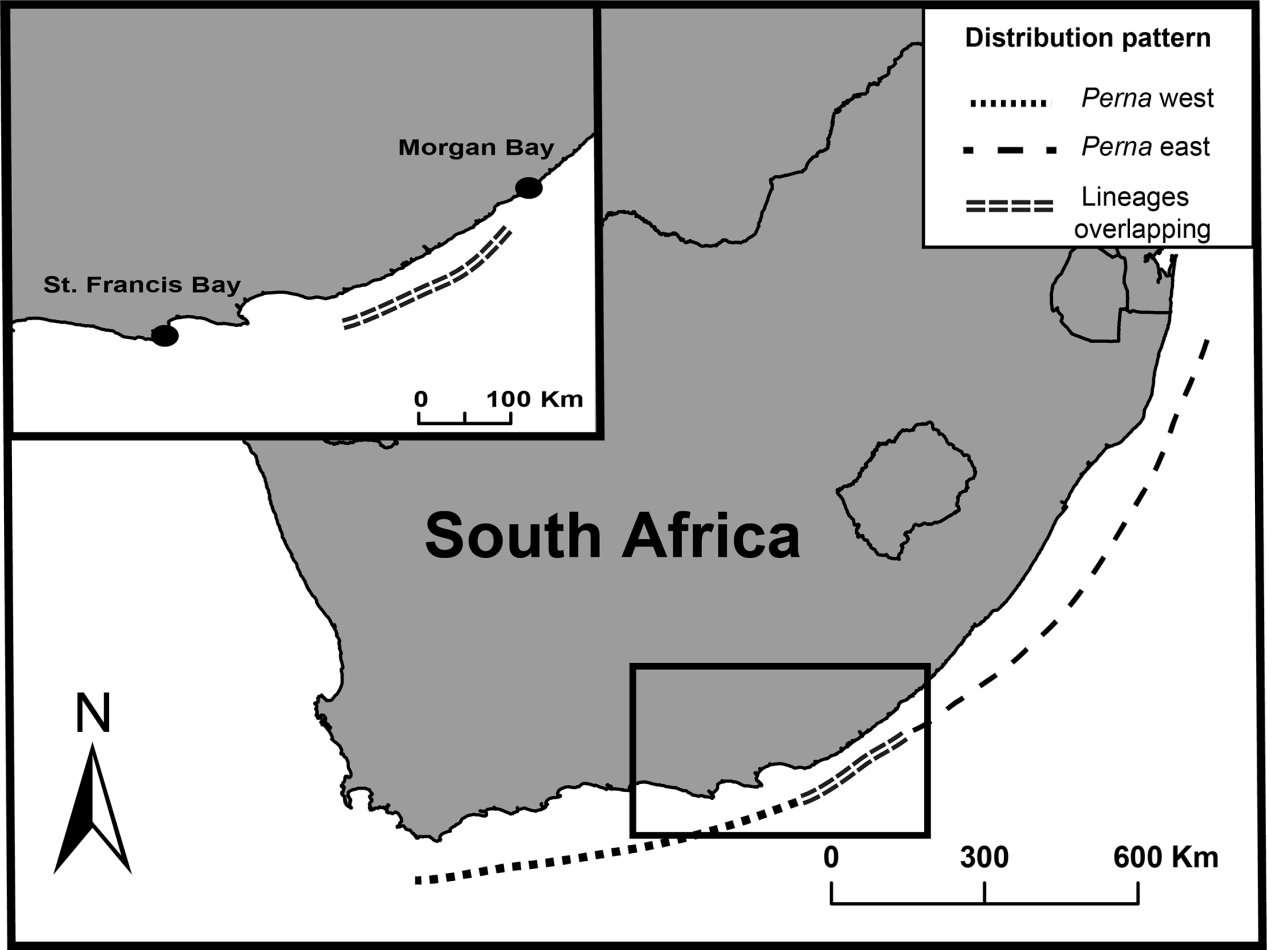
Field sites for *Perna* east (St. Francis Bay) and *Perna* west (Morgan Bay) and the geographic distributions of the two genetic lineages. Note that the study sites fall outside the area of lineage overlap.

*In situ* experiments on the two genetic lineages were performed at two sites outside the region where the two overlap. The sites are c. 370km apart and both are semi-protected rocky shores, with a similar semidiurnal tidal regime and with spring-tide amplitude of about 2m (Fig 1). Unpublished data from *in situ* temperature loggers suggest that the more easterly Morgan Bay is characterized by slightly higher annual average temperatures (18.0°C in air and 18.6°C in water) than Saint Francis Bay (17.6°C in air and 17.9°C in water). The heart rates of animals belonging to the eastern lineage were measured in Morgan Bay (32°42’40”S, 28°20’21”E) from 1 to 2 March 2014. Measurements of western lineage individuals were performed in Saint Francis Bay (34°10’15”S, 24°50’05”E) from 16 to 17 February 2014. Mussel body temperatures were estimated at the same sites for one year (31 January 2014 to 31 January 2015) using biomimetic ‘robomussels’ (see below).

### Temperature measurement

Biomimetic devices (“robomussels”) have been shown to be an effective method of estimating body temperatures in the field [12,13]. Temperature loggers (iButton DS1922L, Dallas Maxim, CA, USA) were embedded in an epoxy resin, encased in mussel shells of about 5–6 cm length and calibrated according to Lima & Wethey [14]. To determine the effectiveness of our biomimetic devices for estimating *P*. *perna* body temperatures, two live mussels (5–6 cm shell length) of each lineage were fitted with a thermocouple and recorded body temperatures were compared with values obtained from six robomussels. The day before this experiment, a small hole (2mm) was drilled in the shell and a K-Type probe connected to a Fluke 54 II was inserted into the hole and sealed with glue. The robomussels and the live mussels with the thermocouple were placed in a container immersed in a water bath and temperature was recorded in both air and seawater. The water bath temperature was increased from 18°C to 40°C or decreased from 18° to 0°C at a rate of 9°C per hour in order to allow the robomussels and the live mussel to be at the same temperature as the water bath. The root mean square error (RMSE) and the Pearson’s correlation coefficient between live mussel and robomussel temperatures were calculated for each medium.

Body temperature was estimated in the field by deploying two robomussels per site next to natural mussel beds in sun and wave exposed areas of the rocky shore where thermal stress is likely to be most severe. Robomussels were deployed using a Z-spar splash zone epoxy glue (Kopper’s Co., USA) [15]. Temperature data, with a resolution of 0.06°C, were collected continuously with a sampling interval of 30 min. Tidal height was calculated every 30 min for each site using free tidal prediction software (Marées dans le monde 4.00, StrassGrauerMarina Softwares). Air and seawater temperature data were determined by identification of the emersion/emersion periods using the tidal height values. The height on the shore was identified by a sharp drop in temperature of ≥3°C in 30 min during summer following [16] and comparison with tide tables. Daily maxima, minima and average temperatures were calculated for each site and the results were compared using a one-way ANOVA with site as a random factor (STATISTICA v. 12, Statsoft). Finally, we calculated the total number of hours and days with temperatures above the average breakpoint limit for heart rate of 29.4°C previously estimated in the laboratory for each lineage of *P*. *perna* [10].

### *In-situ* recording of cardiac activity

Heart rate was measured using a non-invasive technique introduced by Depledge et al. [17] and modified by Burnett et al. [18]. The sensor (CNY-70) consisted of an infrared emitter and phototransistor in a lead-lined package, which blocks visible light. During the low tide period, each sensor was glued onto the shell of an animal of 3–4cm shell length living in the middle of the shore level occupied by mussels and surrounded by other mussels without dislodging the animal. The sensor was glued next to the mid-dorsal posterior hinge area, which corresponds to the position of the heart, and was connected to a recording system placed on the high shore using a 40 m cable that was stabilised by anchoring it at several points along its length. The animals were left undisturbed for 10 min before the start of recording. Signals were amplified (Newshift AMP03), digitized using a portable oscilloscope (PicoScope 2204) and recorded using PicoScope 6 sofware with a sampling rate of 300 S/s. The cardiac activity of each individual was recorded for 30 min intervals, during both daytime and night, under the following conditions: when the animal was completely dry (emersed, E), when initially wetted by the waves (partially immersed, PI), when completely immersed (immersed, I) and when starting to dry just after emersion (partially emersed, PE). As the shapes of heartbeats in the raw signal are very different among individuals, automatic counting can cause mistakes in measuring heart rates [18]. Manual counting can also be questionable, in terms of reliability and practicality [19]. For these reasons, heart rates were estimated using ARTiiFACT software that couples automated peak detection with manual and algorithmic detection of artefacts. The heart rate was analysed in three steps: first, interbeat intervals were automatically extracted from the raw signal using global threshold detection criteria. Second, manual identification of obviously erroneous beats was performed in order to eliminate possible errors due to animal movements, wave action or equipment noise. Third, instantaneous heart beats were calculated from the interbeat interval and averaged per minute. For each individual, heart rate was calculated for five random minutes from the 30 min recording and an average heart rate was computed. Results were expressed as average heart rate for the two individuals measured (HR, beats per minute). Mussel body temperatures during heart rate measurements were estimated from robomussel data for each site.

In order to compare the field results with previous laboratory experiments using animals collected from the same areas [10], a temperature correction was applied. For each temperature condition measured in the field, the laboratory HR was estimated from the Arrhenius equations relating temperature and HR. The calculation was repeated for each laboratory-studied individual (n = 5) and the results were expressed as average heart rate at that temperature. A model I factorial ANOVA was performed on the results with treatment (laboratory/field) as the first factor and condition (emersion day, emersion night, immersion day, immersion night) as a second factor.

## Results

### Temperature measurements

The preliminary laboratory test showed that the average RMSE deviation of robomussels from live mussel body temperatures was 0.5°C. The Pearson’s coefficient indicated a strong correlation between temperature recorded with robomussels and for live animals (r = 0.99 for both lineages and all treatments, n = 6).

**Fig 2:**
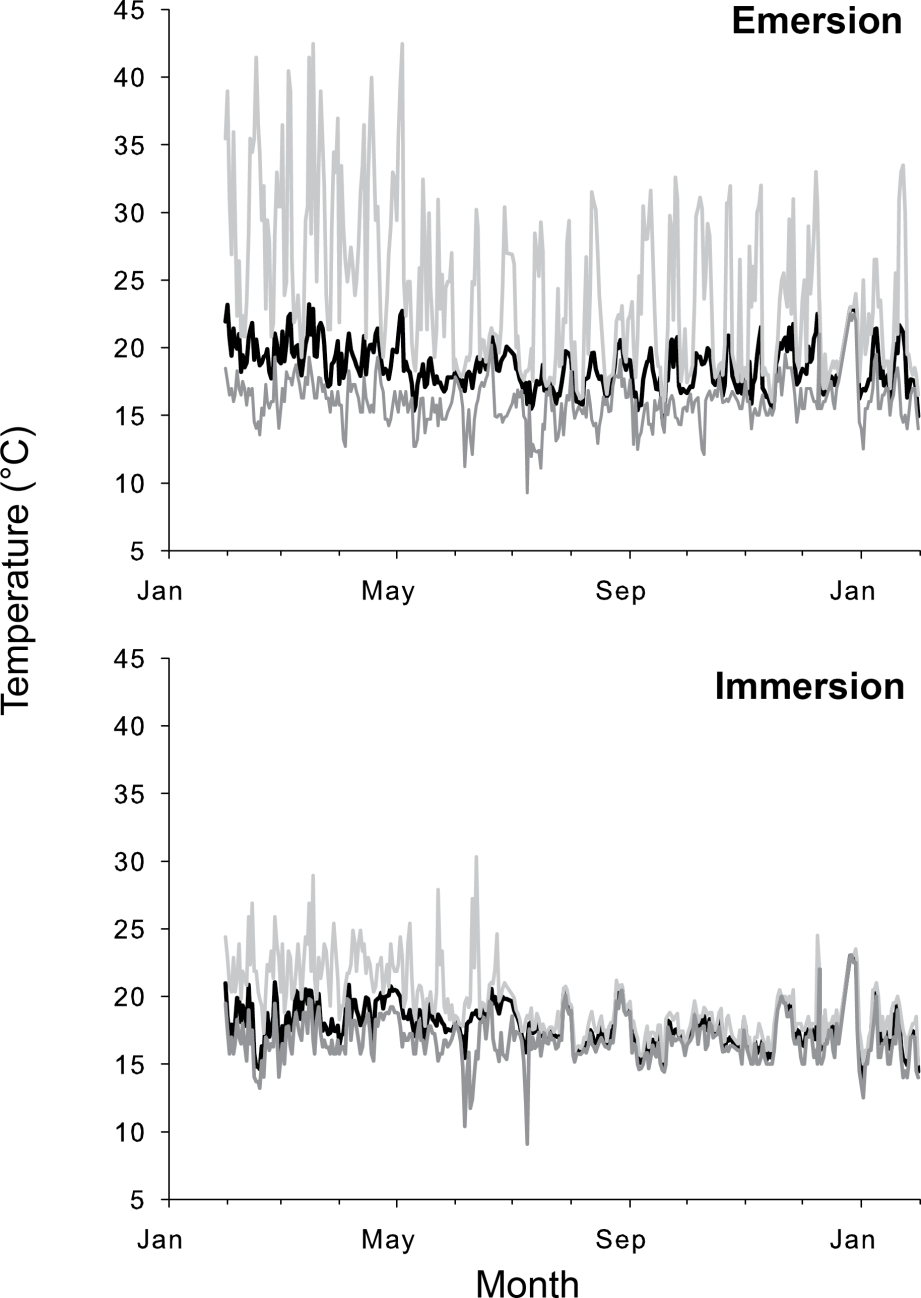
Daily mean (black line), maximum (light grey line) and minimum (dark grey line) temperatures recorded *in-situ* using robomussel data loggers at Morgan Bay during both low and high tide.

**Fig 3:**
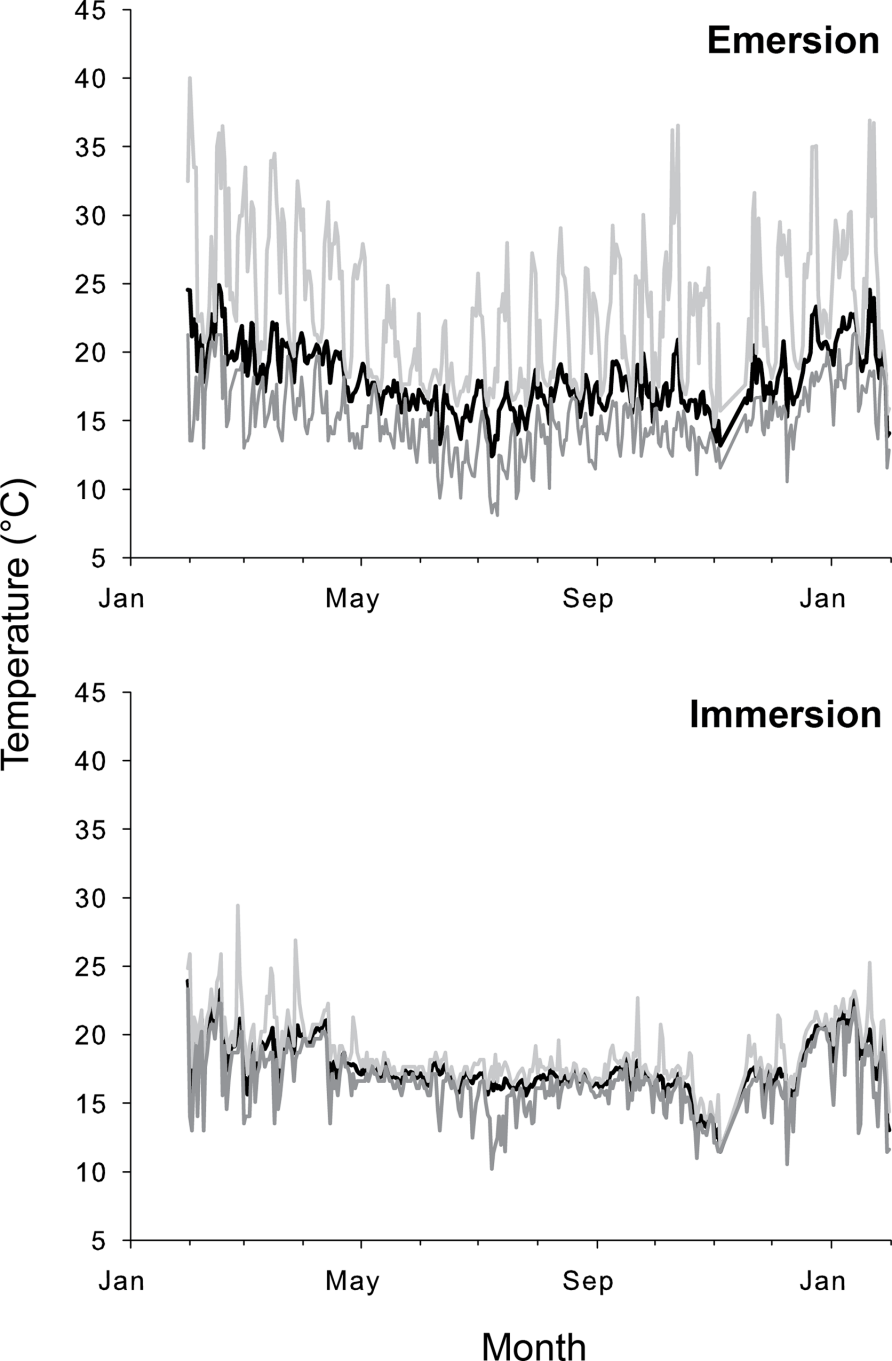
Daily mean (black line), maximum (light grey line) and minimum (dark grey line) temperatures recorded *in-situ* using robomussel data loggers at St. Francis Bay during both low and high tide.

As expected, *P*. *perna* individuals in the field were subjected to large, rapid and cyclic changes in temperature (Figs 2 & 3). During immersion, robomussel temperatures measured in the field varied between 10.2 and 29.4°C in St. Francis Bay and between 9.1 and 30.3°C in Morgan Bay. The temperatures measured during emersion were more variable and ranged between 8.1 and 40.0°C in St. Francis Bay and 9.3 and 42.5°C in Morgan Bay. Temperatures at the two sites differed significantly in aerial daily average, maximum and minimum temperatures (one-way ANOVA, p<0.05 in all cases). In contrast, body temperatures under submerged conditions were significantly different only in the daily average and maximum values (one-way ANOVA, p<0.05) (Figs 2 & 3). During aerial exposure, temperature increased from submerged values by as little as 1°C during winter and as much as 28°C during summer.

During the study period, body temperatures during immersion exceeded the laboratory-determined average physiological breakpoint of 29.4°C on only one day in Morgan Bay and even then for no more than 15 min just after immersion. Over the course of a whole year, however, robomussel temperatures during emersion in Morgan Bay were higher than 29.4°C for 113h (over the course of 80 days and for a maximum of 5 consecutive hours) and higher than 40°C for 6h (over 5 days with a maximum of 30 consecutive minutes). High temperature events in St. Francis Bay were less frequent and less prolonged, with 86h (over 49 days and a maximum of 4 consecutive hours) showing temperatures higher than 29.4°C and 1h (over 1 day) showing values higher than 40°C.

### Heart rate

**Fig 4:**
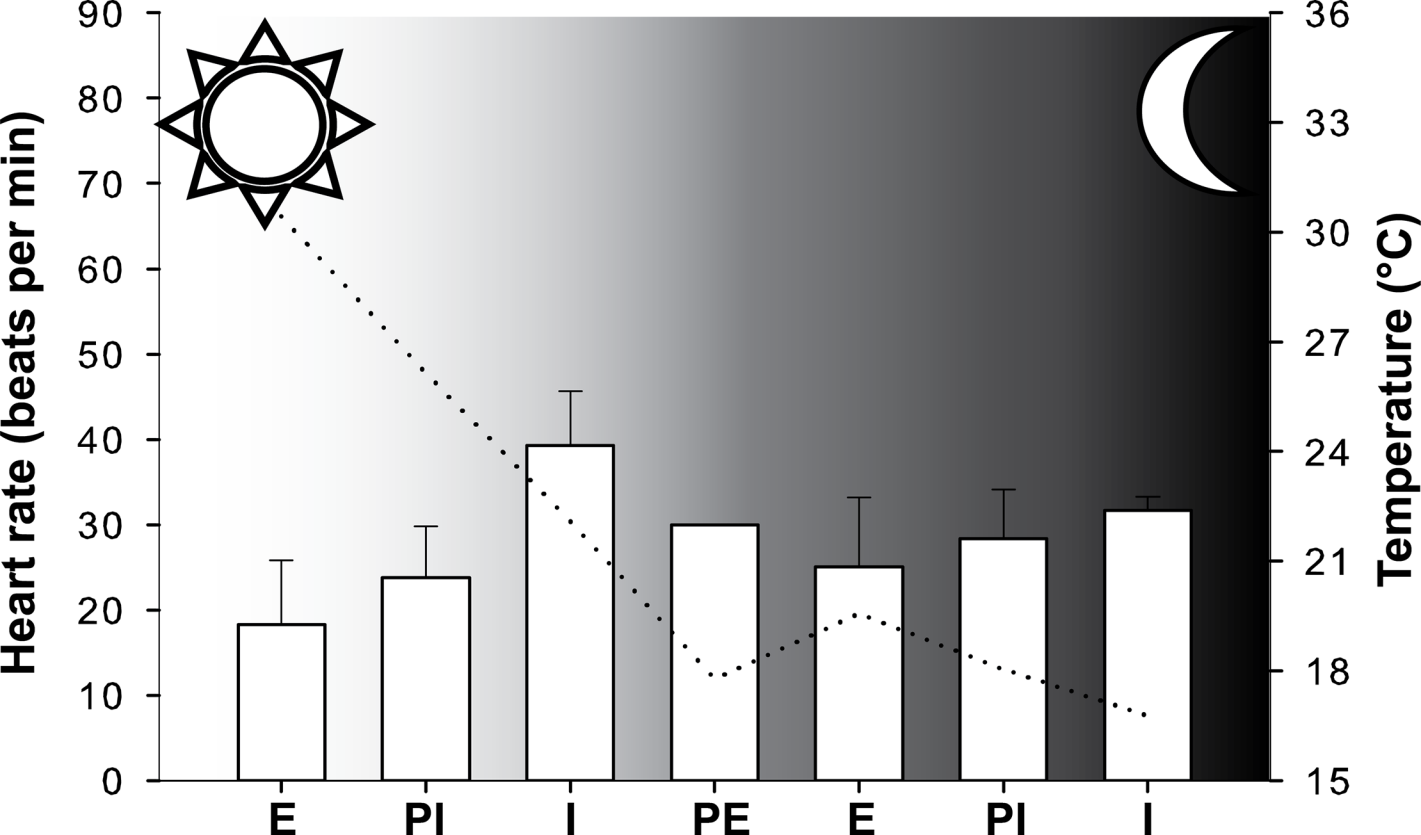
Heart rate variability during a complete tidal cycle measured for *P*. *perna* east individuals (average ± standard deviation, n = 2). The dotted line shows the variation in the average temperature measured during the recording. The sun and moon symbols indicate the day and night periods. E, emersion; PE, partially emersed; I, immersion; PI, partially immersed.

**Fig 5:**
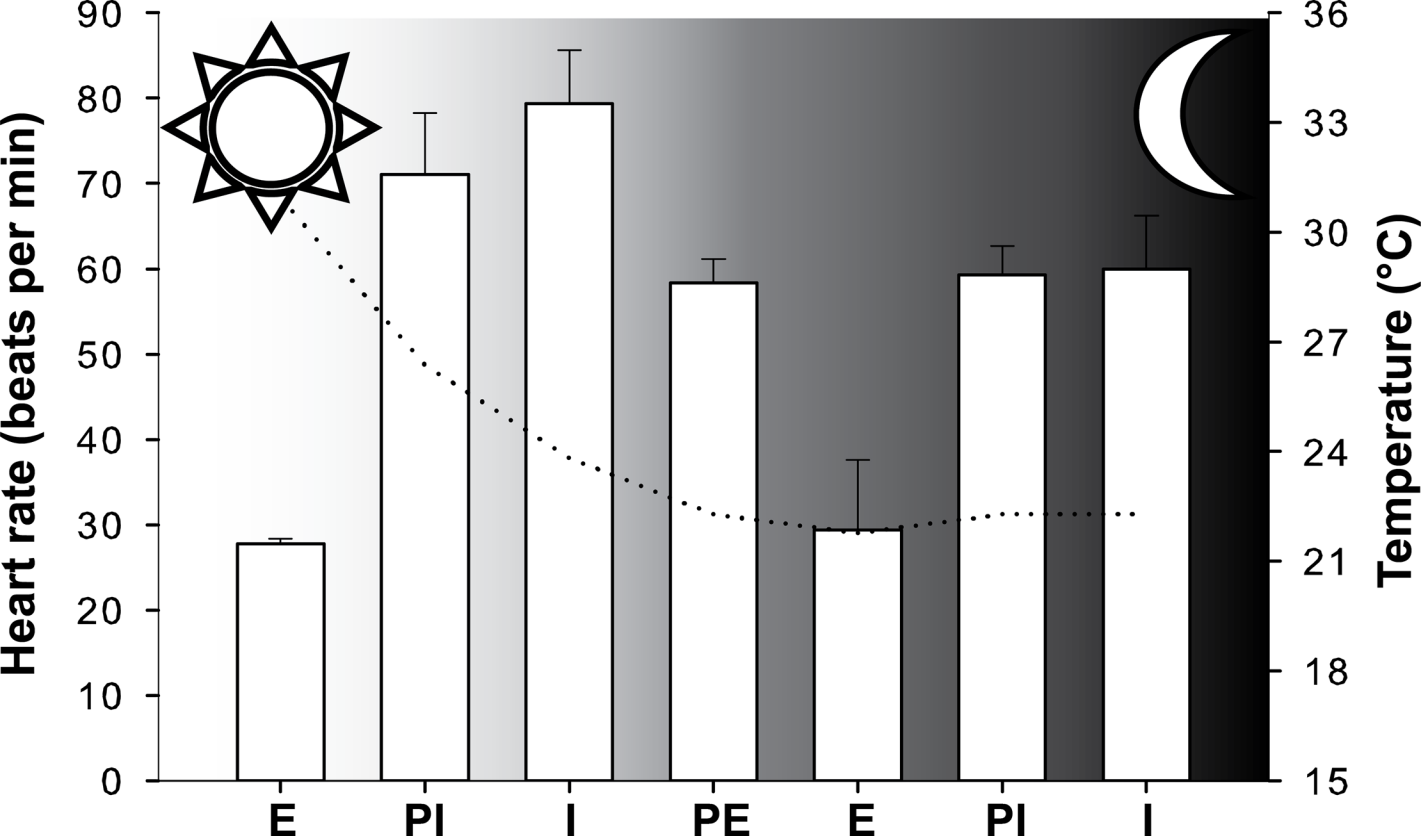
Heart rate variability during a complete tidal cycle measured for *P*. *perna* west individuals (average ± standard deviation, n = 4). The dotted line shows the variation in the average temperature measured during the recording. The sun and moon symbols indicate the day and night periods. E, emersion; PE, partially emersed; I, immersion; PI, partially immersed.

Field recordings showed that the heart rate was strongly affected by the tidal regime and temperature (Figs 4 & 5). Minimum mean values occurred in the day during emersion for both the east and west lineages (18.3 and 27.7 beats min^-1^ respectively). The highest mean HRs were recorded underwater during the daytime for both lineages (39.6 beats min^-1^ for the east lineage and 79.4 beats min^-1^ for the west lineage). The field experiments were conducted on different days and, temperatures were slightly higher in St. Francis Bay (average water and air temperatures 23.2 and 24.6°C respectively during the experimental period) where the western lineage is found, than in Morgan Bay where the eastern lineage occurs (average water and air temperatures of 17.3 and 20.2°C respectively during the experimental period).

Extrapolation of the HR from laboratory-derived Arrhenius plots showed that the laboratory experiments generally showed lower HR values than those measured in the field. HR measured during immersion in the field for *P*. *perna* west was on average 27 beats min^-1^ higher than values calculated from the laboratory results and 6 beats min^-1^ higher for *P*. *perna* east (Table 1). ANOVA analysis comparing HR measured in the field with the extrapolation from the two laboratory protocols (Table 1) revealed that the effect of immersion/emersion was always significant (p<0.05) and that for *P*. *perna* west, field measurements were significantly higher than laboratory measurements (p<0.05). For *P*. *perna* east, HR values were higher in the field during the night but the difference was not significant, possibly reflecting the small sample size.

**Table 1:** Comparison of heart rate (HR) measured in the field and HR extrapolated from the Arrhenius plot calculated from data collected in the laboratory. Values are means ± standard deviation.

	*P*. *perna* west				*P*. *perna* east			
	Field HR	Lab. ramp n°1	Lab. ramp n°2	Temp.	Field HR	Lab. ramp n°1	Lab. ramp n°2	Temp.
**Immersed day**	79.4 ± 6.2	45.3 ± 7.9	45.9 ± 5.3	23.8	39.3 ± 6.3	39.3 ± 4.6	34.7 ± 16.6	22.1
**Immersed night**	59.9 ± 6.3	39.8 ± 7.1	39.6 ± 4.7	22.3	31.6 ± 1.6	23.2 ± 1.7	18.7 ± 8.8	16.8
**Emersed day**	27.8 ± 0.6	36.8 ± 18.8		30.9	18.3 ± 7.5	19.0 ± 5.7		30.4
**Emersed night**	29.4 ± 8.2	28.1 ± 5.0		21.8	25.0 ± 8.1	20.4 ± 7.7		19.6

## Discussion

A major challenge for predicting biological reactions to changing environmental conditions is understanding how multiple stressors interact to affect physiological responses. This is a particular problem in extrapolating from the laboratory to the field as conditions in the field can include unknown factors, particularly the past of an animal, such as its recent experience of stress or its feeding history. Not only can stressors reinforce one another, they can also be cumulative. Temperature and aerial exposure are the most important factors affecting the metabolism and survival of rocky shore species [20], and here we investigated variability in the heart rate of *P*. *perna* individuals over a complete tidal cycle during the stressful summer period, coupled with estimations of individual body temperatures from robomussels deployed over a year.

### *P*. *perna* body temperature

Our laboratory tests confirmed that robomussel loggers are a reliable technique for estimating mussel body temperatures, providing good estimates for intertidal *P*. *perna* [12,14]. Consequently, we can assume that the robomussels deployed in the field were able to provide a good estimation of the real-world body temperatures of individuals exposed to sun and extreme temperature variations.

Although Morgan Bay and St. Francis Bay are relatively close together, separated by approximately 370km, the average, maximum and minimum daily temperatures experienced by mussels were significantly different between the two sites in both air and water. Similar, important *in-situ* temperature variability among intertidal locations has also been demonstrated for the southeast coast of Australia [21]. *P*. *perna* is widespread along thousands of kilometres of the coasts of southern Africa, experiencing a wide range of environmental conditions, but exists as two genetic lineages that occupy different parts of the coast and have different physiological properties [10,11].

Like other rocky shore species, *P*. *perna* in South Africa routinely experience drastic fluctuations in body temperature [16,22] that can cause mortality [23,24]. Data from our robomussels showed that body temperature during the first minutes of immersion can occasionally reach the heart rate breakpoint temperature previously measured for *P*. *perna* in the laboratory [10]. Moreover, the body temperature during emersion can often exceed the breakpoint temperature of 29.4°C and, as found by Zardi et al. [11] for nearby sites, can exceed 40°C, which is close to the upper lethal limits of this species [10,25]. Such extreme conditions during emersion and the first minutes of immersion probably cause extensive mortality especially during summer when the high temperature conditions can be prolonged [11].

### Heart rate variability *in situ*

Our data demonstrate that is possible to monitor the cardiac activity of intertidal mussels directly in the field during both high and low tide. Few studies have measured heart rates *in-situ* using non-invasive techniques during a complete tidal cycle and data are available only for crabs and limpets [1,3,26,27], though the cardiac activity of intertidal mussels has been studied in the field using invasive techniques that require the implantation of electrodes [7,28].

Like the mussel *Mytilus edulis* and the limpet *Patella caerulea* [1,7], both lineages of *P*. *perna* were able to modulate the heart rate rapidly during transitions between immersion and emersion, allowing quick adaptation of the metabolism to the rapid environmental changes that occur in the intertidal. In contrast to intertidal limpets [1,3], mussels seemed unable to maintain high metabolic rates during emersion, especially during the day when temperatures were higher. The important reduction of the heart rate during low tide is probably related to a quiescent condition already documented for *Mytilus* [28]. The diel rhythms seemed less pronounced than tidal rhythms [27,29], but the results for both lineages indicate that the cardiac activity of immersed animals decreased during the night, probably due to a decrease in temperature rather than food availability. In contrast, the heart rate of emersed mussels was slightly higher during the night when desiccation is less of a problem. The absence of a metabolic peak at the beginning of immersion suggests that *P*. *perna* does not demonstrate a metabolic debt after exposure to air and probably the gaping behaviour of this species [30,31] prevents the accumulation of metabolites during emersion [32].

Unfortunately we could not make a statistical comparison of heart rate activity in the two lineages because the environmental conditions differed between the two sites and the number of individuals recorded was low due to the difficulties of weather, wave action and logistics associated with this type of *in-situ* experiment. As in the laboratory [10], the two lineages showed very similar heart rate patterns, while the differences in the actual rates can be explained by the different temperatures each experienced during the experiment. Consequently, metabolic responses to temperature do not offer a good explanation for the geographic separation of the two lineages.

### Comparison between *in situ* and laboratory results

Perhaps more importantly, comparison of the results obtained in the field with those extrapolated from laboratory experiments showed that laboratory measures generally underestimated cardiac activity. Mechanisms causing thermal limits are complex and still largely unknown [33]. Differences between laboratory and *in-situ* physiological rates have been explained by dissimilarities in food availability and feeding rates [34]. In our studies, in order to limit the differences between the two experiments, the laboratory heart rate measurements were performed on animals collected from the same beds as those used in the field experiments, kept in natural seawater and allowed to feed during the experiments.

This study shows that our laboratory experiment generally underestimated mussel heart rates and that this underestimation was particularly severe during immersion. In the case of the western lineage, underestimation was by as much as 75% during daytime immersion when the water temperature was higher. Critically, animals in the field tolerated high temperatures that would be considered lethal on the basis of laboratory experiments. Thus, rather than seeing impaired temperature tolerance as a result of synergistic stressors in the field, we actually recorded unexpectedly improved tolerance of high temperatures. The effect of additional stressors such as desiccation and solar irradiance appears to influence the thermal performance and thermal optima of *P*. *perna*, but in an unexpected way. Animals used for laboratory experiments are generally held under stable conditions prior to experimentation. The increase in HR measured under natural conditions may be an adaptive physiological response to the highly changeable conditions experienced in the field.

## Conclusions

This study focused on the physiological responses of two *P*. *perna* genetic lineages under field conditions to evaluate how they may differ, and, importantly, how field responses may differ from physiological responses measured in the laboratory.

The results indicate that this species lives in areas where it is constantly submitted to strong thermal and desiccation stresses that significantly affect its metabolism. The “cost of living” arising from living permanently close to their thermal limits may be one of the main explanations for intertidal species distributions [35] and mechanisms of sub-lethal thermal sensitivity modulation can be key physiological adaptations for animals subject to frequent thermal stresses.

Overall, the results underline the critical importance of performing *in-situ* experiments on undisturbed animals in order to evaluate the reliability of laboratory experiments as a way of estimating field responses and deriving realistic assessments of the physiology of intertidal species under natural conditions [2]. Laboratory experiments are important tools for understanding how single phenomena act on an animal’s physiology, but real metabolic variability and limits can only be assessed for animals living in the field. One of the most urgent challenges for marine scientists is understanding how climate change will affect the physiology of different organisms [36]. Clearly, our study underlines the need to develop and test techniques that allow the evaluation of the effects of multiple stressors under field conditions if we are to estimate accurately the synergetic effects of climate change drivers on species survival and distributions.
